# Influence of Elongation of Paclitaxel-Eluting Electrospun-Produced Stent Coating on Paclitaxel Release and Transport through the Arterial Wall after Stenting

**DOI:** 10.3390/polym13071165

**Published:** 2021-04-05

**Authors:** Zhanna K. Nazarkina, Boris P. Chelobanov, Konstantin A. Kuznetsov, Alexey V. Shutov, Irina V. Romanova, Andrey A. Karpenko, Pavel P. Laktionov

**Affiliations:** 1 Institute of Chemical Biology and Fundamental Medicine, Siberian Branch, Russian Academy of Sciences, 630090 Novosibirsk, Russia; boris.p.chelobanov@gmail.com (B.P.C.); kostya.kuznetsov.89@inbox.ru (K.A.K.); irin-romanova@yandex.ru (I.V.R.); lakt@niboch.nsc.ru (P.P.L.); 2 Lavrentyev Institute of Hydrodynamics, Siberian Branch, Russian Academy of Sciences, 630090 Novosibirsk, Russia; alexey.v.shutov@gmail.com; 3 Meshalkin National Medical Research Center, Ministry of Health of the Russian Federation, 630055 Novosibirsk, Russia; andreikarpenko@rambler.ru

**Keywords:** paclitaxel, drug-eluting stents, polycaprolactone, electrospinning

## Abstract

It was previously shown that polycaprolactone (PCL)-based electrospun-produced paclitaxel (PTX)-enriched matrices exhibit long-term drug release kinetics and can be used as coatings for drug-eluting stents (DES). The installation of vascular stents involves a twofold increase in stent diameter and, therefore, an elongation of the matrices covering the stents, as well as the arterial wall in a stented area. We studied the influence of matrix elongation on its structure and PTX release using three different electrospun-produced matrices. The data obtained demonstrate that matrix elongation during stent installation does not lead to fiber breaks and does not interfere with the kinetics of PTX release. To study PTX diffusion through the expanded artery wall, stents coated with 5%PCL/10%HSA/3%DMSO/PTX and containing tritium-labeled PTX were installed into the freshly obtained iliac artery of a rabbit. The PTX passing through the artery wall was quantified using a scintillator β-counter. The artery retained the PTX and decreased its release from the coating. The retention of PTX by the arterial wall was more efficient when incubated in blood plasma in comparison with PBS. The retention/accumulation of PTX by the arterial wall provides a prolonged drug release and allows for the reduction in the dose of the drugs in electrospun-produced stent coatings.

## 1. Introduction

Recently we have shown that electrospun (ES)-produced paclitaxel (PTX)-enriched matrices are suitable for vascular stents coating ) [1,2]. Drug-eluting stents (DES) were designed to minimize neointima growth after angioplasty [3]. PTX is one of the most commonly used drugs for in-clinic peripheral DES coatings. The cytotoxic effect of PTX is mediated by its binding to cellular microtubules with nanomolar specificity, promoting the formation of discrete microtubule bundles in cells and cell division arrest [4]. PTX was shown to inhibit smooth muscle cell (SMC) proliferation and migration and prevent neointima formation after angioplasty [5]. The clinical trials confirmed the efficacy of PTX in reducing restenosis rates [6]. According to Kuno et al., PTX-eluting stents and balloons did not increase short-term mortality vs. balloon angioplasty [7]. PTX-eluting stents did not show a significant increase in long-term mortality vs. balloon angioplasty. However, PTX-coated balloons showed a significant increase in long-term mortality compared to balloon angioplasty and bypass. In contradistinction to PTX-coated balloons that deliver a large drug dose over a short time period, stents with lower drug doses provide prolonged PTX delivery after implantation. This observation suggests that the dose of PTX is important. Indeed, a pre-clinical study in a pig model showed a significant dose-dependent inhibition of neointimal hyperplasia [8]. A high PTX dose (187 µg/stent) resulted in a better suppression in neointimal formation, an increase in mean luminal diameter, and an inflammatory reduction in comparison with lower doses (0.2 and 15 µg/stent). Histological changes including focal neointimal acellular material bridging the gap between the medial wall and strut sites, and necrosis of the medial wall cells with associated calcified deposits and medial wall hemorrhage were observed and correlated with the applied drug dose. The fluoropolymer-based PTX-eluting peripheral stent, Eluvia (Boston), was designed to provide controlled and sustained paclitaxel release over 12 months. Eluvia stents showed promising outcomes in short femoropopliteal lesions [9]. Despite the low PTX concentration of 0.167 µg/mm^2^ in stent surface area (the drug dose density is 18 times less than that in the ZILVER-PTX stent), aneurysm formation detected in 8% of cases was thought to be attributable to paclitaxel. Likely, PTX release during a long period has a negative effect, and new strategies for DES design are demanded.

Paclitaxel is a small compound with a molecular weight of 853.9 Da. Due to its hydrophobicity and low solubility, PTX binds strongly to tissue protein elements and remains in the tissue under the stent struts for some time after release [10]. The solubility of PTX depends on the drug’s crystallinity and the composition of the surrounding solution. The aqueous solubility of the amorphous form of PTX is 31.6 μg/mL and the solubility of the dihydrate crystalline form is 0.36 μg/mL [11], while toxic concentrations of PTX against SMC vary in the nanomolar range [5]. The binding of PTX to plasma proteins increases its solubility in blood plasma 20–60 fold [12]. The concentration of PTX bonded to the artery in static conditions in phosphate-buffered saline (PBS) is 30 times higher than that in solution [13]. Under steady-state loading conditions, PTX remains in the subintimal space and significantly accumulates in the adventitia. The planar diffusivity for PTX exceeded the transmural diffusivity by two orders of magnitude [13]. Despite its fast planar PTX diffusion in the arterial wall, there is an obvious overdose of PTX on the coating of the stent struts. Considering the toxicity of PTX concentrations against SMC, its dosage seems orders of magnitude higher than necessary. As these PTX doses in DES are generally accepted and required for cytotoxic effects, the currently used delivery approaches are not completely acceptable. Thus, new and more efficient delivery approaches are required. We should also take into account the fact that stenting procedures lead to the stretching of the vessel wall and consequently to the reorganization of the structure of the arterial tissue, as well as the changing of the distance between PTX-binding sites and their effective concentration. As these factors affect the distribution and accumulation of PTX in the arterial wall, they must be considered when calculating the PTX dose.

The materials used for DES coating need to have mechanical characteristics and biological properties similar to natural vessels. Coating techniques have an influence on these characteristics and drug release kinetics [14]. Drug coatings can be applied to metallic vascular stents using different techniques: Ultrasonic coating [15], dip-coating [16], spray-coating [3], the air-brush technique [17], electrohydrodynamic jetting, plasma-treated coating, electrotreated coating [14], and electrospinning (ES) [18]. The ES technique is widely used in regenerative medicine and drug delivery. Two ES products (esophageal stent and heart valve prostheses) were granted to FDA-approved clinical studies [19]. The advantages of coating stents with electrospun-produced matrices are the following: (1) drug release can be controlled through the structure of polymer fibers; (2) the drug is equally distributed across the surface of the entire stented section of the artery; (3) the coating distributes the load of the strut-wall contact region to the entire surface area occupied by the stent; (4) like an endothelial coating, it performs a barrier function, preventing tissue debris from entering into the blood, as well as eliminating direct contact between blood cells and the damaged tissue [2].

Synthetic materials produced from polycaprolactone (PCL) are widely used in medicine owing to the PCL biocompatibility, good mechanical properties, and ease of manufacturing [20]. The materials produced from pure PCL possess hydrophobicity, a low degradation rate, and a low cell compatibility. The incorporation of HSA into the PCL matrices increases the stiffness and biological properties of such matrices [1,21]. A previous study has shown that matrices ES-produced from a solution of PCL, human serum albumin (HSA), 1,1,1,3,3,3-hexafluoroisopropanol (HFIP), and dimethyl sulfoxide (DMSO) with a low PTX dose are most suitable for stent coatings [1]. These matrices do not exert additional stress on the stent beams, they exhibit two-phase PTX release kinetics providing a prolonged PTX release, and they can potentially maintain a therapeutic PTX concentration in the arterial wall. The stents coated with 5%PCL/10%HSA/3%DMSO/PTX demonstrated a weaker neointima growth, a slower increase in the blood flow rate, and the absence of arterial wall thickness in comparison with bare-metal stents after in vivo installation in a rabbit iliac artery [2]. Manipulations performed during the stent installation can affect the integrity and properties of the stent’s coatings. In vitro and ex vivo experiments with stents coated with nonpolymeric PTX using dip-coating demonstrated a significant postdeployment decrease in PTX levels caused by the handling of the stent before the mounting, cracking, and flaking of the paclitaxel film, distortion of the stent during crimping, blood washout, etc. [8]. This means that the chemical and mechanical characteristics of the coatings have great importance. The stent’s diameter increases twofold during installation, thus elongating its coating. The integrity of the individual fibers and the matrix as a whole is important for stent coatings. Fiber disruption can lead to a change in the rate of PTX release. Here, we have studied the influence of matrix elongation on its structure and PTX release kinetics, as well as the retention of PTX by the stretched arterial wall.

## 2. Materials and Methods

### 2.1. Production of ^3^H-Labeled Paclitaxel

Tritium-labeled paclitaxel (^3^H-PTX) was synthesized by thermoactivated tritium exchange, as described earlier [22], using PTX from (ACROS Organics, Geel, Belgium). ^3^H-PTX was purified after labeling using reversed-phase chromatography, and the radioactivity of the samples was evaluated as reported earlier [1].

### 2.2. Fabrication of the Matrices by Electrospinning

The electrospinning (ES) solutions were prepared using stock solutions of 9% of PCL and 1% of HSA (Sigma-Aldrich, St. Louis, MI, USA) in 1,1,1,3,3,3-hexafluoroisopropanol (HFIP, Sigma-Aldrich, USA). The HSA concentration in matrices was 10% (given as a weight percentage (*wt.*/*wt.*) of total matrix weight). PTX dissolved in HFIP or DMSO (Sigma-Aldrich, USA) was added to the ES blend to achieve a final concentration in the matrix of ~0.46 µg/cm^2^, which corresponds to 0.36 µg per 10 mm diameter disk, ~0.785 cm^2^. In addition, 3% of DMSO (*v*/*v*) was added to the solution of polymers. To obtain more uniform fibers, PCL/SRL ES solution contained 0.1 mM of triethylamine (TEA). ^3^H-PTX was diluted with unlabeled PTX to provide at least 25,000 cpm/cm^2^ (or 20,500 cpm per disk). To produce ^3^H-PTX-containing matrices, a custom-made ES device with an airproof chamber and exhaust HEPA filter was used, equipped with a Spellman SL 150 (30 kV, Spellman, Brockton, MA, USA) power supply. Matrices of 110–150 µm in thickness were prepared using a drum collector (4 cm in diameter and 5 cm in length) under the following conditions: A feed rate of 1 mL/h; 20 cm capillary-collector distance; 26.5 kV voltage; collector rotation speed of 300 rpm; temperature of 23–25 °C; humidity of 25–35%. After fabrication, matrices were removed from the collector, dried in a vacuum under 10 Pa for 12 h, and stored in sealed zip-lock polyethylene containers at 4 °C.

### 2.3. Fabrication of PTX-Eluting Stents

ES solutions were prepared as described in Section 2.2. A polished stainless-steel rod (1.0 mm in diameter and 30 mm in length) with a previously installed metal stent (min/max diameter 2.0/3.5 mm; Angioline, Novosibirsk, Russia) was used as an electrode [2]. A coating of 125–150 µm was applied onto the stent under the conditions described in Section 2.2. Each stent required 0.23 mL of the polymer solution. After fabrication, the coated stent was removed from the collector, vacuum-dried, and stored as mentioned above. Before being installed to a balloon catheter, the coated stents were put on a rod with a diameter of 1 mm with a conic junction from 1 to 1.5 mm. The stent was pushed from this end onto the balloon catheter preliminary folded as an “asterisk.” After the installation, using a syringe manometer, the stent was additionally rolled in for sealing on the delivery device simultaneously with the evacuation of the balloon catheter.

### 2.4. SEM Analysis

The microstructures of the matrices were studied via scanning electron microscopy (SEM), as described earlier [21]. To evaluate the structure after elongation, the mechanical device described in [23] was used. The matrices and stents were fixed on a sample stand using double-sided carbon tape, sputter-coated with 10 nm of gold/palladium, and analyzed using a scanning electron microscope EVO 10 (Carl Zeiss AG, Oberkochen, Germany) at an accelerating voltage of 10 kV.

### 2.5. Modeling of Uniaxial Deformation of the Matrices

The finite element simulation of the mechanical loading was carried out using the commercial code MSC.MARC. The electrospun material was modeled using the classical constitutive equations of finite strain elasto-plasticity with linear isotropic hardening [24]. This model was implemented into MSC.MARC as a user-defined material; the implementation was based on the numerical algorithm from [25]. The initial dimensions of the sample were 10 mm × 10 mm. During active loading, an axial displacement of 10 mm was prescribed. During unloading, the sample shortened to 17 mm. Both the loading and unloading were carried out in 250 time-steps. The total Lagrangian description of kinematics was activated in MSC.MARC. A mesh of 10 × 10 finite elements using a quadratic approximation of the geometry and displacements was used with full integration.

### 2.6. PTX Release Kinetics from Intact and Twofold Elongated Matrices

To evaluate PTX release, 10 mm diameter disks were excised from the matrices via die cutting and placed in the wells of a 48-well plate. Then, 400 µL of PBS or EDTA-stabilized blood plasma (BP) was added to each well. The work was approved by the Local Ethical Committee of the Center of Personalized Medicine ICBFM SB RAS (No 1, 15.01.2016). The plate was sealed with a film (Microseal^®^ ‘B’ PCR Plate Sealing Film, adhesive, Bio-Rad, Hercules, CA, USA) to prevent drying, followed by incubation on a Titramax 1000 shaker (Heidolph, Schwabach, Germany) at 37 °C and with a platform rotation speed of 200 rpm. Two series of PTX release kinetics were studied. The matrices were incubated in PBS or BP for 20 min, 60 min, 3 h, 9 h, 27 h, 3 days, 9 days, and 27 days without or with medium replacement. In the second case, the supernatant was removed at each time point, fresh PBS or BP was added, and the disks were incubated until the next time point. The radioactivity of the supernatants was measured in duplicate, as described in Section 2.1.

To obtain twofold elongated matrices, they were fixed in a special device and force was applied. After stress relaxation, the matrices were cut into discs used for studying PTX release. The distance from the clamps was 10–15% of the sample length. The linear sizes of the matrices were measured after stretching, and the amount of PTX per disk was calculated.

### 2.7. PTX Release from Stent through Arterial Wall 

Laboratory Chinchilla rabbits with an age of 6.5 ± 0.2 months and bodyweight of 4.5 ± 0.6 kg were used in this work. All manipulations of the rabbits complied with the European Convention of laboratory animal protection [26] and were approved by the Local Ethical Committee of NMRC MH RF (NCT02255188 from 16 January 2014). After the autopsy, the iliac artery was excised and washed with PBS. The stent mounted on balloons (SINUS coronary stents 3.5 × 18 mm, Angioline, Novosibirsk, Russia) was introduced in the artery and was positioned so that the artery completely covered the stent. The stent, covered with the artery, was placed in a through-hole 2.5 mm in diameter drilled into a 5 mL tube (Corning, NY, USA) close to the bottom. The balloon was dilated with a basic COMPAC inflation device. Then, 1 mL of PBS or BP was added in a tube at time-point zero. The samples were incubated at 25 °C and aliquots of 200 µL were taken from different time intervals (20 min, 60 min, 3 h, 9 h, and 27 h) to calculate the radioactivity at each time point, and an additional 200 µL of fresh medium was introduced in a tube with subsequent incubation until the next time point.

### 2.8. Diffusion Flow and Effective Diffusivity of PTX through the Arterial Wall

To estimate the thickness of the arterial wall of the rabbit iliac artery, a series of measurements were performed using a RIFTEK RF651 contactless micrometer (Riftek, Belarus) in 10 planes before and after the removal of the artery from the stent.

The diffusion flux (j) and effective diffusivity (D) of PTX through the arterial wall were calculated as [27]

j = Δm/SΔt; D = −j ∂x/∂C(1)

where ∆m is the mass of PTX transferred through a surface with area S in time ∆t, and C is the concentration.

### 2.9. Statistical Processing of Data

Microsoft Excel 2010 was used to handle and process the experimental data. Statistical analysis was performed using the Statistica 7.0 package (StatSoft Inc., Tulsa, OK, USA).

The difference factor f1 and similarity factor f2 were calculated as [28]

(2) $$1=\left\{\left[\overset{n}{\underset{t=1}{\sum }}\left|R_{t}-T_{t}\right|\right]/\left[\overset{n}{\underset{t=1}{\sum }}R_{t}\right]\right\}*100,$$

(3) ff2=50∗log1+1n∑t=1nRt−Tt2−0.5∗100

*R_t_* and *T_t_* are the cumulative percentages released at each of the selected *n* time-points of the reference and test product, respectively.

## 3. Results

### 3.1. Preparation and Characterization of the PTX Matrices 

Tritium-labeled PTX (^3^H-PTX) was synthesized via thermoactivated tritium exchange, as described earlier [22]. A RP-HPLC-purified ^3^H-PTX preparation with a specific radioactivity of ~0.3 mCi/mL was obtained. The compound was homogenous according to the TLC data, as it was detected as a single spot on the autoradiograph with the expected Rf corresponding to unlabeled PTX. ^3^H-PTX was combined with unlabeled PTX to reach a dose of ~0.46 μg/cm^2^ of PTX and a radioactivity of ~25,000 cpm/cm^2^.

Based on previous data, matrices produced from 5%PCL/10%HSA/3%DMSO/PTX are most suitable for stent coatings, exhibiting long-term PTX release kinetics [1]. To clarify whether the matrix content will interfere with the efficacy of PTX release after matrix elongation, three different matrices (5%PCL/PTX, 5%PCL/10%HSA/PTX, and 5%PCL/10%HSA/3%DMSO/PTX) were prepared using electrospinning on a drum collector and tested in the current study.

The physicochemical properties of the matrices were described earlier [1]. The tensile strength of these matrices varies from 4 to 10 MPa; the matrices exhibit a typical two-phase curve with an extended region of plastic deformation (from 8–10% to 260–300%). The mechanical properties of the produced matrices provide elongation without failure during stent expansion, as well as a low residual load in the stent coating after its installation [1].

**Figure 1:**
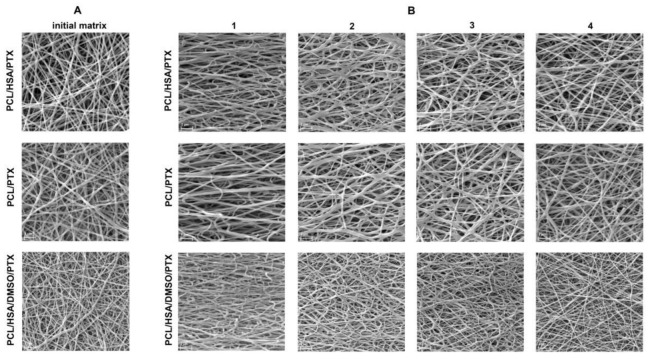
(**A**) SEM images of the intact matrices. (**B**) SEM images of different areas of the elongated matrices. The numbers represent the regions in the various positions of the elongated matrix (see Figure 2A).

According to SEM data, all matrices were composed of randomly oriented fibers (Figure 1A). The SEM reveals the smooth surface of the fibers. The composition of the ES solution influenced the structure of the matrices. The addition of DMSO in the ES solution resulted in a decrease in fiber diameter. The decrease in fiber diameter for matrices containing DMSO was also shown for PCL-based matrices containing sirolimus [23]. 

### 3.2. The Influence of Matrix Elongation on the Fiber Structure

**Figure 2:**
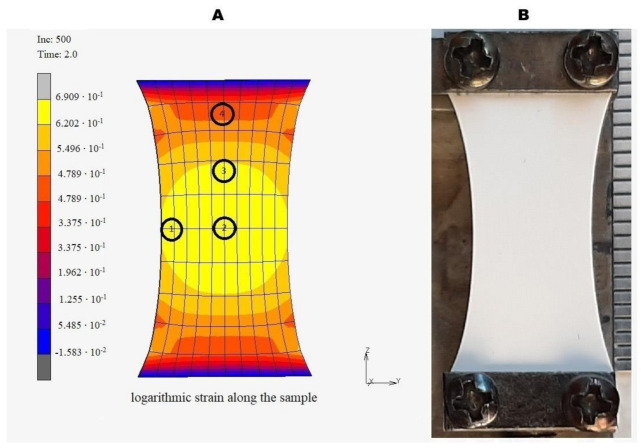
(**A**) A graphical representation of the sample deformation with control points. The numbers represent the regions of the matrix in the various positions of the extended matrix. (**B**) The device used for the elongation of matrices with the elongated sample.

The installation of the vascular stents includes a twofold elongation of the matrices covering the stents. Thus, to study PTX release from the twofold elongated matrices, they were fixed in a tensile device, and force was applied to obtain a twofold uniaxially elongated matrix. To study the effect of deformation on the structure of the matrices depending on the position between the clamps and considering Poisson’s deformation, we used a device allowing us to expand and fix the matrix with an SEM camera, as described earlier [23]. The uniaxial elongation of the matrix results in the heterogeneous deformation of the sample (Figure 2) and could thus interfere with the structure of fibers located in different regions of the sample. Four areas of the matrices were studied via SEM and are marked in Figure 2A. It was shown that the two-fold matrix elongation resulted in an orientation of the fibers along the applied force vector, without forming fiber breaks and without changing fiber diameter (Figure 1B), despite the deformation of the sample. An unequal deformation of the matrices depending on the position was demonstrated. The maximum strain was observed in the center area (position 1). However, the matrix near the clamps subjected to minimal stretching resembles the intact matrix (position 4). The absence of broken fibers, according to SEM data, means that all tested matrices are able to withstand elongation when installing the stent.

Uneven matrix deformation suggests a necessity to introduce coefficients during the study of PTX release if the discs of the same area will be cut out from different areas of the deformed matrix. To evaluate the applicability of our common approach for the analysis of PTX release (from discs of the same diameter), a uniaxial deformation of the matrices was modeled, and the change in the area of matrices in different locations of the elongated matrix was estimated (Figure 2A). The material parameters employed in the simulation are summarized in Table 1. Note that the material parameters are chosen such that the external force becomes zero upon unloading (elastic springback). The finite element simulation shows a good correspondence with the real experiment in terms of the final geometry. The kinematic parameters of the sample after unloading are listed in Table 2. The modeling showed that, despite the significant heterogeneity of the matrix deformation due to edge effects, the change in the area at the control points varies from +37.3% to +43.7%, i.e., the difference between the control points is bounded by 6.4%. This means that the PTX amount in the disk does not significantly depend on the position of the disks in the matrices and the disc position does not affect the release of PTX. 

### 3.3. Influence of Matrix Elongation on PTX Release

**Figure 3:**
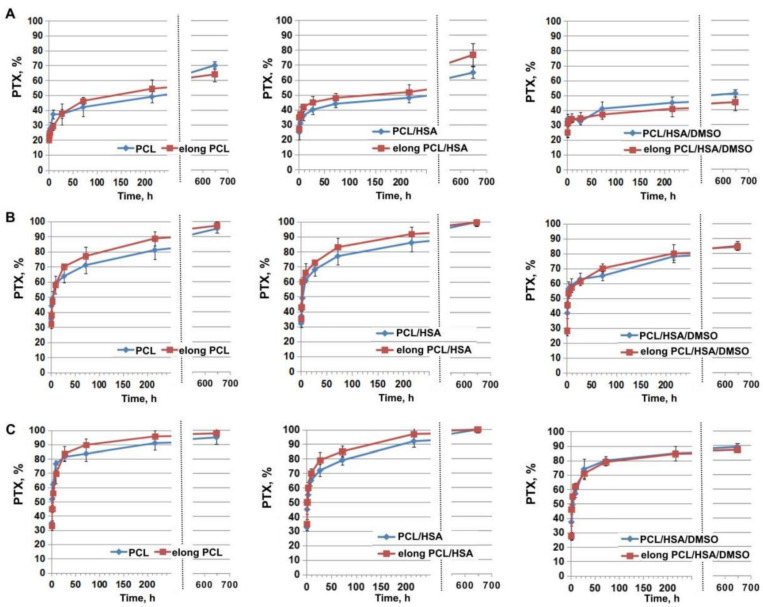
The kinetic curves of PTX release from intact and elongated matrices. The illustrations show incubation of the matrices in (**A**) PBS without medium replacement; (**B**) blood plasma (BP) without medium replacement; (**C**) BP with medium replacement.

Considering the twofold elongation of the matrices covering vascular stents taking place during stent installation, the effect of matrix deformation on drug release from ES-produced matrices was studied. We compared the kinetic curves of PTX released from intact and twofold elongated matrices obtained from 5%PCL/PTX, 5%PCL/10%HSA/PTX, and 5%PCL/10%HSA/3%DMSO/PTX solutions (Figure 3). The data obtained demonstrated that deformation has practically no effect on the kinetics and completeness of drug release from the matrices. The nature of the kinetic curves and the end-points coincide with good accuracy. The most important thing is that the matrix of the composition 5%PCL/10%HSA/3%DMSO/PTX, which is optimal for the manufacture of coatings for bare-metal stents [1], does not change properties, i.e., the kinetics of PTX released from the elongated matrix is similar to that of the intact matrix, regardless of the matrix composition. To confirm this observation, difference (f1) and similarity (f2) factors were calculated (Table 3). Fit factor values demonstrated the similarity of the kinetic curves of PTX released from initial and elongated matrices.

A similar effect was observed earlier for sirolimus [23]. The twofold elongation of the matrices barely changed the kinetic curves of SRL release from the matrices with BP replacement.

### 3.4. The Effect of the Arterial Wall on the Rate of PTX Release

**Figure 4:**
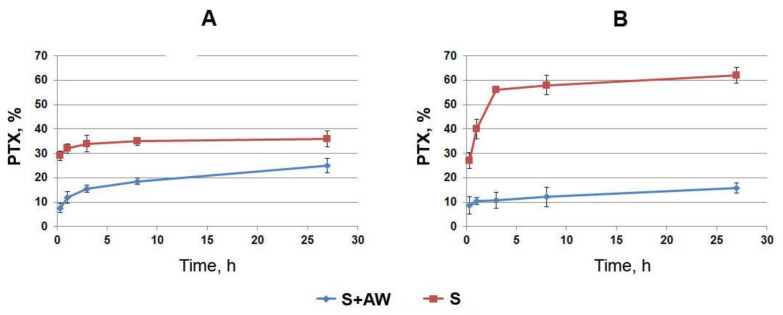
The kinetics of PTX penetration through the arterial wall. The PTX-eluting stents covered with the arterial wall (S+AW) or noncovered PTX-eluting stents (S) were incubated in (**A**) PBS or (**B**) BP.

**Figure 5:**
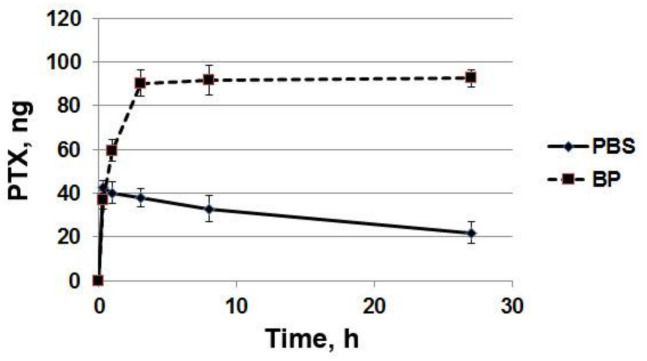
The kinetics of PTX accumulation in the arterial wall. The data are presented as a difference between PTX released from the stent coating and PTX passed through the arterial wall and detected in the surrounding medium.

The effect of the arterial wall (rabbit iliac artery) on the rate of paclitaxel release from stent coatings was studied. The kinetics of PTX diffusion through the arterial wall from stents coated with 5%PCL/10%HSA/3%DMSO/PTX with paclitaxel at a dose of 0.46 μg/cm^2^ is presented in Figure 4. Coated stents were placed on balloon catheters, inserted into the rabbit’s freshly obtained iliac artery, and the release of the PTX through the arterial wall was studied by measuring the radioactivity of the PTX released into the surrounding PBS or human blood plasma. As shown in Figure 4, the arterial wall effectively retains the drug. It was found that the arterial wall slows down the release of paclitaxel into the solution 3–4 times during the first few hours and 2–2.5 times during the day. After 24 h of incubation of the stent covered with the arterial wall, more than half of the PTX released from the coating was retained. About 25% of the PTX potentially released from the matrix into the BP diffused through the wall of the iliac artery, while the rest of the drug was retained in the wall. In this case, the release into the BP (an imitation of tissue fluid) was slower for PTX than PBS. The data on the accumulation of PTX in the arterial wall were calculated as the difference between PTX released from the stent coating and PTX passed through the arterial wall and detected in the surrounding medium (Figure 5). The data demonstrate the loss of part of the PTX from the arterial wall in PBS and the constant growth of PTX accumulation in the wall in BP.

The PTX release into the PBS seems to be connected with the accelerated diffusion of soluble PTX-binding components like human serum albumin present in the arterial wall compared to their diffusion into BP. HSA binds paclitaxel with a binding constant of 1.43 × 104 M^−1^ [29]. The plasma albumin concentration normally ranges from 35 to 50 g/L [30], and 60% of the albumin is located in the interstitial and intracellular space [31]. Thus, HAS could be considered as a potential candidate as a PTX transporter in PBS but not in BP.

The data on PTX delay in the arterial wall when incubated under conditions similar to those in vivo [2] are in favor of the long-term maintenance of the sub-cytotoxic drug concentration in the arterial wall and make it possible to reduce the dose of drugs introduced into the coating.

The PTX dose used in this study was significantly lower than the absorption threshold dose (estimated in the order of 0.2 μg/cm^2^/h^1/2^). This means that a significant fraction of PTX is absorbed by the arterial wall. According to our estimates, the thickness of the arterial wall of the rabbit iliac artery is approximately 0.34 ± 0.05 mm. These data allow us to estimate the diffusion flow (j) and effective diffusivity (D) of PTX through the arterial wall. The estimated diffusion flow through the arterial wall was 2.3 pg/c/cm^2^. The effective diffusivities in the arterial tissue were estimated as 1.20 and 1.11 µm^2^/s in PBS and BP, respectively.

## 4. Discussion

Previous studies have shown that matrices produced from 5%PCL/10%HSA/3%DMSO/PTX are suitable for bare-metal stent coatings and provide a prolonged PTX release. They could theoretically maintain a therapeutic PTX concentration in the arterial wall [1] and they are efficient, as confirmed by in vivo studies [2]. The installation of the vascular stents entails a twofold elongation of the matrices covering the stents. To mimic PTX release from the stent coating, which is evenly elongated during the radial twofold increase in the balloon diameter, we used uniaxially elongated matrices produced from the same material. This type of deformation differs from the deformation during stent implantation, whereupon uniform deformation of the tube walls occurs, but it can simulate the influence of deformation on fiber location and structure. Twofold deformation does not lead to fiber breaks or strong modification of the matrix in terms of porosity and structure. Finite element modeling showed that, despite the significant heterogeneity of the matrix deformation due to edge effects, the PTX amount in the disk did not significantly depend on the position of the disks in the matrices, and thus, the disc position can be disregarded in the experiment. Our data confirm that deformation has practically no effect on the kinetics and completeness of drug release from matrices. The coatings containing 5%PCL/10%HSA/3%DMSO/PTX display two-phase PTX release kinetics. The first stage (during the first day) is characterized by rapid PTX release, the second (up to 27 days) by slow PTX release. The rapid PTX release in the first stage ensures the delivery of the drug to the neointima and prevents inflammation. According to the literature, the inflammatory and thrombotic phases are maximal during the first hours after stenting; a proliferative phase has the highest activity of SMC at 7 days post-stenting [32]. Thus, the rate of PTX release from elongated matrices should be sufficient to minimize neointima growth after angioplasty. The absence of fiber damage, according to SEM data, means that all tested matrices are able to withstand elongation when installing the stent, which is confirmed by optic control of the stenting procedure (Figure S1).

Drug-coated devices are widely used for the treatment of peripheral artery disease. Recent studies demonstrate the advantages of using PTX-eluting stents compared to PTX-coated balloons [7,33]. It should be noted that the concentrations of PTX used for coating the balloons and commercially available PTX-eluting stents are an order of a magnitude higher than those used in the current study. PTX has nanomolar affinities to microtubules [4], and toxic concentrations of PTX for SMCs are also at a nanomolar level [5]. Thus, devices and coatings able to maintain effective PTX concentrations in arterial tissue for a long time with minimal drug entry into the bloodstream are still required. The stents coated with 5%PCL/10%HSA/3%DMSO/PTX using a PTX dose of 0.46 µg/cm^2^ contain significantly less PTX than TAXUS and ZILVER stents do, but they prevent neointima growth in vivo [2]; however, its comparison with clinically used polymer-coated stents (e.g., Eluvia), including the study of medial layer injury, inflammation, and fibrin deposition, makes sense. According to our estimates, the total concentration of PTX eluted from the ES coating in the arterial wall is in the range of 0.1–1 µM. Considering PTX toxicity, this dose of PTX should be sufficient for the reliable inhibition of neointima growth. Tzafriri et al. predicted that the penetration of strongly retained drugs is dependent on the surface concentration and that the biological effect may become dominant due to the presence of drug gradients in the target tissue [34]. Based on its binding potential, PTX (Bp > 40) is referred to as a strongly retained drug with a significant concentration-dependent arterial transport. The dose intensity threshold (the minimal dose that causes an effect without toxicity increases) of PTX was estimated to be of the order of 3 μg/cm^2^/h^1/2^. The tissue binding capacity, binding potential, and drug diffusivity determine the drug absorption by the tissue. Sub-threshold doses of PTX will lead to the absorption of a significant part of the eluted drug by the tissue and its retention in the artery. An increase in PTX dose until a threshold is reached will lead to an increase in drug absorption, penetration, and occupancy of tissue PTX-binding sites. A dose above the absorption threshold will result in the formation of a pool of free PTX near the lumen, which will be washed away. The TAXUS-PTX stent can provide a PTX dose of 20.3 μg/cm^2^/h^1/2^, implying significant drug washout. 

The PTX dose used in our study was significantly lower than the absorption threshold dose. This means that a significant fraction of PTX was absorbed by the arterial wall, and entry into the bloodstream should be minimal. The estimated diffusion flow through the arterial wall was 2.3 pg/c/cm^2^. The median effective diffusivities in the arterial tissue were estimated as 1.20 and 1.11 µm^2^/s in PBS and BP, respectively. The diffusion coefficients measured at the starting points of incubation and near the end of the experiment differ by almost 10-fold. This fact could be connected with the excessive amount of PTX quickly entering the artery wall just after the stent’s installation, which exceeds the number of PTX-binding centers possessing reasonable constants for fast PTX complexing (like HSA-PTX Ka). Three or 12 h after DES installation in the arteria, practically all PTX released by the coating remained bound to the artery wall (Figure 5), demonstrating the redistribution of PTX between binding sites and the involvement of low-affinity sites, whose numbers rise with PTX concentration in the arterial wall. The medium diffusivity values found in our study are approximately 5.5 times higher than the values obtained for the penetration of PTX in vivo in the rat carotid artery and ex vivo in the calf carotid artery by Lovich et al., which were estimated as 0.2 µm^2^/s [35]. The data differ from the diffusivity of PTX in the blood serum by approximately 20-fold (diffusivity amounts of 20.3 µm^2^/s in the serum and 76.6 µm^2^/s in protein-free buffer). It should be noted that in the current study, the artery was supported with stents, and its diameter was increased at least twofold, simultaneously decreasing the thickness of the artery wall and increasing its linear dimensions. Of course, the packing density of the biomolecules and their supramolecular structure inside the artery wall are drastically decreased. It was shown that the planar diffusivity for PTX exceeded the transmural diffusivity by two orders of magnitude [13], suggesting a layered/planar location of PTX binding sites. The distancing of PTX binding sites and reorganization of the arteria wall immediately after the stenting procedure, as well as the subsequent adaptation of artery wall components to new conditions and diffusion of blood plasma molecules, must interfere with PTX deposition in the artery wall. Despite individual molecular mechanisms, the data obtained suggest that the migration of PTX is determined by the equilibrium between the dissociation and binding of the PTX to the components of the arterial wall. A PTX content of the covering of ~0.46 μg/cm^2^ provides a practically total absorption of PTX by the arterial wall with minimal loss of PTX thanks to its diffusion through the arterial wall. The estimated concentration of PTX in the arterial wall is two orders of magnitude higher than the cytotoxic effect and seems to be stable for a long time. It should be noted that, in contrast to the experimental conditions where diffusion into the blood was eliminated by the installation of the balloon, diffusion into the blood could also occur in vivo. To increase the efficacy of drug elution coatings and vectorize drug delivery, a special layer preventing drug loss in circulation is needed.

## 5. Conclusions

Earlier, we demonstrated that stents coated with 5%PCL/10%HSA/3%DMSO/PTX installed in the rabbit iliac artery showed weaker neointima growth, a slower increase in blood flow rate, and the absence of thickness of the arterial wall in comparison with bare-metal stents [2]. The installation of vascular stents involves a twofold increase in stent diameter and, therefore, stretching of the matrix covering the stents. Matrix deformation during stent installation does not lead to the formation of fiber breaks, and the kinetics of PTX released from the elongated matrices is identical to that of the initial matrices. Ex vivo studies have shown that the arterial wall is simultaneously elongated during stenting; nevertheless, it prevents the cross-wall penetration of PTX into surrounding tissues. The accumulation of PTX in the arterial wall promotes the long-term maintenance of sub-cytotoxic drug concentrations in the wall and allows us to reduce the dose of drugs introduced into the coating.

## Figures and Tables

**Table 1:** The material parameters of the elasto-plastic model of Simo and Miehe.

Elasticity Modulus	Poisson’s Ratio	Initial Uniaxial Yield Stress	Linear Isotropic Hardening Coefficient
1 [dimension of stress]	0.45 [-]	0.05 [dimension of stress]	0.225 [dimension of stress]

**Table 2:** The parameters of the sample after elongation.

	Point Number			
	1	2	3	4
Stretch in the axial direction	1.883	1.996	1.851	1.594
Stretch in the transverse direction	0.729	0.720	0.766	0.872
Change of area [×times]	1.373	1.437	1.417	1.390

**Table 3:** A comparison of release curves using fit factors f1 and f2. A comparison is given of the kinetic curves of SRL release from the intact and the twofold elongated matrices.

**Matrices Incubated in PBS without Any Medium Replacement**			
**Matrix Type**	**5%PCL/PTX Elongated**	**5%PCL/10%HSA/PTX Elongated**	**5%PCL/10%HSA/3%DMSO/PTX Elongated**
5%PCL/PTX	f1 = 11.9 f2 = 64.7	-	-
5%PCL/10%HSA/PTX	-	f1 = 9.6 f2 = 67.6	-
5%PCL/10%HSA/3%DMSO/PTX	-	-	f1 = 7.31 f2 = 73.8
**Matrices Incubated in BP without Any Medium Replacement**			
**Matrix Type**	**5%PCL/PTX Elongated**	**5%PCL/10%HSA/PTX Elongated**	**5%PCL/10%HSA/3%DMSO/PTX Elongated**
5%PCL/PTX	f1 = 6.8 f2 = 65.2	-	-
5%PCL/10%HSA/PTX	-	f1 = 8.2 f2 = 60.1	-
5%PCL/10%HSA/3%DMSO/PTX	-	-	f1 = 4.2 f2 = 75.0
**Matrices Incubated in BP with Medium Replacement**			
**Matrix Type**	**5%PCL/PTX Elongated**	**5%PCL/10%HSA/PTX Elongated**	**5%PCL/10%HSA/3%DMSO/PTX Elongated**
5%PCL/PTX	f1 = 6.5 f2 = 64.7	-	-
5%PCL/10%HSA/PTX	-	f1 = 6.4 f2 = 65.7	-
5%PCL/10%HSA/3%DMSO/PTX	-	-	f1 = 5.4 f2 = 68.4

## Supplementary Materials

The following are available online at https://www.mdpi.com/article/10.3390/polym13071165/s1; Figure S1: An electrospun-coated stent installed onto balloon catheter before (A) and after (B) balloon expansion. SEM image of coating after balloon expansion (C).
