# Supplementary material for: Influence of Elongation of Paclitaxel-Eluting Electrospun-Produced Stent Coating on Paclitaxel Release and Transport through the Arterial Wall after Stenting
Source: Polymers (Basel). 2021 Apr 5;13(7):1165. doi: 10.3390/polym13071165 (PMC8038586; doi:10.3390/polym13071165)
Supplement: Supplementary file 1 [file polymers-13-01165-s001.zip › supplementary file.docx]

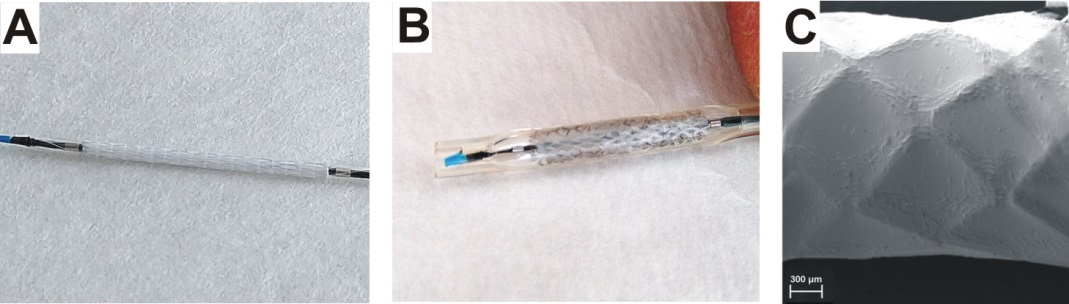


**Figure S1.** An electrospun coated stent installed onto balloon catheter before (A) and after (B) balloon expansion. SEM image of coating after balloon expansion (C).
